# Malnutrition in Patients with Acute Stroke

**DOI:** 10.1155/2011/167898

**Published:** 2011-12-27

**Authors:** Stella D. Bouziana, Konstantinos Tziomalos

**Affiliations:** First Propedeutic Department of Internal Medicine, Medical School, AHEPA Hospital, Aristotle University of Thessaloniki, 54636 Thessaloniki, Greece

## Abstract

Stroke is a devastating event that carries a potential for long-term disability. Malnutrition is frequently observed in patients with stroke, and dysphagia contributes to malnutrition risk. During both the acute phase of stroke and rehabilitation, specific nutritional interventions in the context of a multidisciplinary team effort can enhance the recovery of neurocognitive function. Early identification and management of malnutrition with dietary modifications or specific therapeutic strategies to ensure adequate nutritional intake should receive more attention, since poor nutritional status appears to exacerbate brain damage and to contribute to adverse outcome. The main purpose of nutritional intervention should be the prevention or treatment of complications resulting from energy-protein deficit. This paper reviews the evaluation and management of malnutrition and the use of specialized nutrition support in patients with stroke. Emphasis is given to enteral tube and oral feeding and to strategies to wean from tube feeding.

## 1. Introduction

Stroke is the third leading cause of mortality in developed countries after coronary heart disease and cancer [1]. In 2008, a stroke prevalence of 7,000,000 patients has been estimated for the US with an average of one patient dying from stroke every 4 min [1]. Across European countries, stroke incidence varies between 100 and 700 events per 100,000 inhabitants [2]. The incidence of stroke is predicted to increase over the next 5–10 years by 12% in the general population and by 20% in low-income subjects [3]. Mortality rates during the first 30 days after stroke and at 1 year are 20 and 30%, respectively. The large majority (65–85%) of strokes in the Western world are ischemic whereas hemorrhagic strokes, which are less frequent, are more disabling [4]. Between one-third and one-half of patients with hemorrhagic stroke do not survive and only 10–20% of them regain functional independence [5].

Even with optimal acute management, more than 30% of stroke survivors will have severe disability after stroke and 20% will require institutional care at 3 months [1]. Long-term disability represents the most frequent poststroke complication with 50% of patients suffering from hemiparesis and 30% being unable to walk without assistance creating an enormous socioeconomic burden [1, 2].

Malnutrition is frequently observed in patients with acute stroke and during the rehabilitation period. Malnutrition is associated with poor outcome in these patients [6, 7]. We review the prevalence, consequences, and management of malnutrition in patients with stroke.

## 2. Definition of Malnutrition

The term “malnutrition” represents an important preventable complication of acute stroke and is used to describe a large number of nutritional abnormalities. It is characterized by long-standing negative imbalance in both energy and protein intake and requirements, with metabolic requirements exceeding nutritional intake [8, 9], leading to altered body composition and impaired biological function [10].

## 3. Prevalence of Malnutrition

The prevalence of malnutrition after stroke varies widely among published reports [11]. It is estimated that about fifth of patients with acute stroke are malnourished on admission [11]. In a small study in geriatric patients with severe stroke, 56.3% were malnourished at some point during a hospital stay of >3 weeks [8]. On the other hand, in poststroke patients residing in an infirmary, the prevalence of malnutrition was 61% [12]. In a recent systematic review of 18 studies, the frequency of malnutrition ranged from 6.1 to 62% [9]. Differences in the timing of assessment, stroke type (ischemic versus hemorrhagic), comorbid medical conditions, and stroke complications may have contributed to this large variability [8, 9]. However, a substantial proportion of the variation may also be attributed to the heterogeneity of nutritional assessment methods [8, 9]. The prevalence of malnutrition rises with increased length of hospitalization and with decreased functional improvement during rehabilitation [8, 13]. In a studyon 104 patients with acute stroke, protein-energy malnutrition was present in 16.3% on admission, and this rate increased to 26.4% by day 7 and to 35% at day 14 in those who remained hospitalized [8].

## 4. Risk Factors for Malnutrition

On admission, the presence of chronic diseases, polypharmacy, eating difficulties, and functional disability is associated with increased risk of malnutrition, particularly in elderly patients [14]. Diabetes mellitus and a history of stroke increased the risk for malnutrition on admission by 58 and 71%, respectively [8, 12]. In contrast, location or type of stroke, paresis of the dominant arm, socioeconomic status, and education were not significantly associated with malnutrition [8, 12]. Interestingly, deficiency of micronutrients such as B vitamins, vitamin D, antioxidant vitamins (A, C, and E), and zinc [2, 11] appears to contribute to vasculature changes in the brain; moreover, they appear to increase the risk of stroke and cognitive impairment in the elderly (Table 1). However, how these factors are causally interrelated remains poorly understood [11].

Dysphagia is a major risk factor for malnutrition in patients who suffer a stroke. A prospective studyon 49 stroke patients showed that dysphagia and tube-feeding were both strong predictors of malnutrition on admission into a rehabilitation hospital [12, 15]. Malnutrition may develop as a consequence of dysphagia if nutritional intake is substantially reduced in relation to requirements over the course of days or weeks.

Even in patients without dysphagia, inadequate nutritional intake for a prolonged period of time, particularly of proteins, also increases the risk for developing malnutrition, highlighting the importance of closely monitoring nutritional intake [15–17]. Besides dysphagia, factors that contribute to poor nutritional intake include reduced level of consciousness, poor oral hygiene, depression, reduced mobility, and arm or facial weakness [13]. Poststroke depression reduces appetite and has a deleterious influence on recovery of daily living activities. Stroke patients are often prescribed antidepressants, and the prevention of xerostomia induced by these medications should not be overlooked in order to prevent feeding difficulties [8, 18]. It has been estimated that 10 days of bed rest in healthy older adults decreases muscle protein synthesis by 30% and leg lean mass by 6% resulting in 16% reduced muscle strength [1]. Poor mobility or inactivity might also impair insulin sensitivity, which affects glucose-dependent energy metabolism and decreases insulin-induced anabolic stimulation. Patients with hemiplegia may not be able to maintain their head or body in an upright position and may have to eat with the nondominant arm. Patients with acute stroke also often experience fatigue and this causes difficulties with eating. They could stop eating before they have satisfied their hunger, as they need to rest or even fall asleep. If patients eat and drink too little in relation to their needs, this can worsen fatigue and result in undernourishment [19]. Visual, speech, and language difficulties hinder adequate communication about food needs and preferences. Cognitive deficits also limit patients' ability to carry out the activities required to eat a meal thus increasing the risk for malnutrition [8]. The importance of stroke severity in determining the risk for malnutrition is highlighted by the finding that patients with hemorrhagic stroke are significantly more likely to develop malnutrition than those with ischemic stroke [12]. Finally, women are more susceptible than men to undernourishment because they appear to eat less and suffer strokes at an older age. Accordingly, screening for malnutrition should be more thorough in women with stroke, and management of malnourished women should be instituted promptly [19].

Except dysphagia and inadequate nutritional intake, the increased metabolic demands during recovery also increase the risk of malnutrition [11, 20]. Furthermore, older age, poor family or nursing care, no early stage rehabilitation, presence of malignancy, and history of severe alcoholism can contribute to poor nutritional status and dehydration [8, 18]. Preexisting malnutrition can also increase the risk for worsening nutritional status [15]. Notably, almost 90% of the stroke patients are older than 65 years, and the consequences of acute stroke may lead to additional nutritional problems [21].

## 5. Malnutrition as a Risk Factor for Adverse Outcomes

There is ample evidence that malnutrition is associated with poor outcome in patients with both ischemic and hemorrhagic stroke [6, 12, 22]. Protein and energy malnutrition (PEM) on admission influences mechanisms of ischemic brain injury and impairs recovery [7]. PEM alters the expression of plasticity-associated genes that are associated with recovery mechanisms after global ischemia [7]. PEM can also induce changes in hippocampal plasticity-associated proteins, suggesting that PEM may induce abnormalities in structure, function, and plasticity of hippocampal fibres [17]. Following global ischemia in experimental models, PEM intensifies the expression of trkB and GAP-43 protein in the hippocampus, indicating both an increased stress response and hyperexcitability in the hippocampal circuitry [17, 23].

Malnourished patients with stroke experience more intense stress reactions, show higher rates of pressure ulcers and urinary tract and respiratory infections, and have longer duration of hospitalization and higher mortality rates [20]. In a recent large epidemiological study in 21,884 patients admitted for stroke, mortality after 5 years of followup was higher in the underweight group [17]. Among 2,194 poststroke patients followed up in the Feed Or Ordinary Diet (FOOD) trial, undernourished patients were more likely to develop pneumonia, other infections, and gastrointestinal bleeding. Undernourished patients also had higher mortality rates during a median follow-up period of 196 days [12].

Low serum levels of protein and albumin are markers of malnutrition and are associated with impaired functional status, poor outcome, and higher mortality [12]. However, their role as a marker of malnutrition remains controversial because stroke, as a critical illness, might promote inflammation, which in turn increases energy expenditure and promotes muscle catabolism resulting in decreased levels of circulating proteins. Therefore, it is frequently unclear whether low protein levels in a patient with stroke are due to malnutrition or inflammation [10]. However, in patients with acute stroke, lower serum albumin concentration predicted mortality during hospitalization and the need for institutional care [12]. In young patients with ischemic stroke, serum prealbumin (transthyretin, PA) was also an independent predictor of clinical outcome [24].

Serum levels of vitamins A, E, and C are frequently reduced in patients with acute stroke on admission and decrease further during hospitalization [2]. These reduced levels might reflect the presence of malnutrition or represent the result of increased oxidative stress during acute stroke [25]. Low levels of these vitamins have been associated with larger cerebral infarctions, functional decline, and higher mortality rates [2].

Dehydration can also potentially worsen the ischemic process by increasing hematocrit level and blood viscosity and by decreasing blood pressure. Dehydration increases the risk for stroke recurrence, and patients with stroke and high plasma osmolality levels on admission had poorer survival at 3 months [15].

## 6. Assessment of Nutritional Status

Establishing a patient's nutritional status is not always a straightforward task because there is no universally accepted definition of malnutrition or a gold standard for nutritional status assessment [8]. In addition, nonnutritional metabolic stroke consequences can mimic signs of malnutrition and hamper the detection of true malnutrition [9]. Indicators of malnutrition include serum levels of albumin, prealbumin, and transferrin; total lymphocyte count; body weight and body mass index; triceps skinfold thickness; midarm muscle circumference [8]. Inherent flaws with triceps skinfold measurement include low sensitivity and intra- and interobserver variability. Body weight, body mass index, and midarm muscle circumference also have low sensitivity and specificity whereas lymphocyte count and serum levels of albumin, prealbumin, and transferrin are affected by the presence of inflammation. Clinicians should be aware of the strengths and limitations of each of the markers used to assess malnutrition, and it might be preferable to use a combination of markers instead of a single one [9]. An initial assessment of nutritional status will greatly assist in developing a suitable nutritional plan, and occasional reassessments can help guide any changes in the plan that become necessary [26].

Obtaining a detailed nutrition history, including food intake and recent weight history, is another important part of nutritional assessment. If cognitive function limits the patient's ability to provide an accurate history, nutrition professionals should seek this information from family members [8].

## 7. Management of Malnutrition

Both animal and human studies showed that protein synthesis is suppressed and oxidative stress is exacerbated in the brain after acute stroke [2]. Nutritional care appears to have beneficial effects on plasticity mechanisms that are important for recovery after brain ischemia [17]. Nutritional interventions can also enhance the efficacy of stroke rehabilitation through their positive influence on physical and mental functioning [27]. Due to a loss of muscle and fat mass in patients with stroke, nutritional strategies should provide specific and adequate nutritional supplementation to prevent prolonged hospitalization, poor functional outcome, and death [2].

The decision on how to feed a patient with acute stroke should be made shortly after hospital admission. If the gut is functional and there are no other contraindications, enteral feeding is the preferred feeding method [8]. Swallowing function should also be assessed, ideally by a speech-language pathologist [8]. For this purpose, most clinicians use an array of items including pudding, thin and thickened liquids, soft food items, and items that require mastication. Initial volumes usually range from 5 to 10 mL and, if swallowed successfully, larger boluses with similar rheologic characteristics are administered. The patient's performance on each material should be the guide for advancing to larger volumes. This observational aspect of the clinical swallowing evaluation should not be underestimated because selective tolerance of soft foods and thin liquids is predictive of the need for feeding-tube placement [28]. In dysphagic patients, the duration of nutritional support depends on the severity of dysphagia (Table 2).

Poststroke weight status and body composition can change and body weight should be measured regularly during rehabilitation. Nutritional interventions should be targeted at avoiding weight loss and be carried out using a diet adapted to the swallowing ability [29, 30]. Feeding protocols are described in clinical practice guidelines and these protocols should be part of standard of care [31, 32]. Consulting with a clinical dietitian is also important for nutritional assessment and for developing a monitoring plan [26]. Age, obesity, severity of stroke, presence of infection and comorbidities, medications, mobility, and activity levels affect caloric requirements, necessitating frequent reassessment by nutrition clinicians. Indirect calorimetry is the gold standard for determining caloric requirements but is not routinely available. Currently, no single formula to calculate nutrition requirements has been validated in the stroke population [8]. Interestingly, the conventional wisdom that stroke patients are initially hypermetabolic has been challenged over the past decade. Total energy expenditure (TEE) as determined by continuous indirect calorimetry was low during the first 5 days in patients with stroke admitted to intensive care unit [26]. Moreover, caloric requirements might decrease during hospitalization because of reduced mobility [8]. Overall, the following daily nutrition intakes are recommended for clinically stable patients, which are in the subacute stroke phase and have normal renal function: daily protein intake >1 g/kg in order to achieve a carbohydrate/protein ratio <2.5; energy intake ≥25 kcal/kg in nonobese subjects to maintain body weight and <25 kcal/kg in obese subjects to maintain a carbohydrate/protein ratio <2.5 [2].

Accumulating data suggest that adequate protein intake is an important aspect of the nutritional management of patients with stroke. The importance of brain protein synthesis for neuronal survival has been documented, and an epidemiological study showed higher risk for neurological disorders in healthy subjects with low protein intake [33]. In addition, experimental studies showed that protein synthesis is suppressed in the ischemic penumbra [2]. Moreover, the impaired glucose utilization in ischemic brain neurons renders amino acids as an alternative source for the aerobic production of energy in patients with stroke. A recent study aiming at defining optimal nutritional strategies revealed that selective supplementations such as those including essential amino acids may have advantages in preserving muscle metabolic integrity and function [34]. However, essential amino acids are less palatable than intact protein, and data on their safety are limited. Among specific essential amino acids, excessive uptake of methionine should probably be avoided because it converts to homocysteine, which is potentially atherogenic [35]. Another study in patients with acute stroke showed that improvement of neurocognitive deficits correlated positively with dietary protein intake and negatively with the carbohydrate/protein intake ratio [2]. An individualized energy- and protein-supplementation diet during hospital stay also improved health-related quality of life and grip strength after 3 months [14].

In patients with severe dysphagia, enteral nutrition (EN) with a nasogastric tube or through percutaneous endoscopic gastrostomy (PEG) is the treatment of choice [15]. A percutaneous endoscopic jejunostomy (PEJ) tube is the preferred option for patients with pancreatic disease, gastrointestinal tract obstruction, or frequent episodes of aspiration [36]. A jejunal tube can also be placed through a previous gastrostomy (percutaneous endoscopic gastrojejunostomy (PEGJ)), which however requires a longer tube increasing the risk of displacement [32]. When a PEG, PEJ, or PEGJ cannot be performed, a surgically placed gastrostomy or, less often, jejunostomy tube is required [8]. The greater the severity of neurological injury, the more likely is that enteral feeding tubes will be required [8]. Patients typically receive this nutritional support therapy for less than 6 weeks although some may rely on EN throughout their lives [37]. EN is cost-effective, maintains or improves nutritional status, and reduces the risk for malnutrition-related complications [20, 31, 37]. In patients with acute stroke and dysphagia, protein and energy intake was higher in patients receiving enteral tube feeding than in those on a regular or dysphagia diet [38]. In contrast, EN is not appropriate for patients who are able to meet their nutritional requirements orally.

Feeding with both nasogastric tube and PEG is effective for early nutritional support [39]. However, if there are contraindications to enteral feeding such as nonfunctioning gut and insufficient coverage of nutritional needs by EN or if the placement of nasogastric tube and PEG is not feasible, parenteral nutrition can be provided [8, 40]. Compared with parenteral nutrition, EN shows physiological advantages, is less expensive, and is associated with fewer complications [20]. The risk of tube-feeding-related aspiration can be reduced by frequent clinical examination, monitoring of residual volumes, and elevation of the head of the bed. Nasogastric tubes are easy to insert, allow for bedside removal if swallowing function later improves, allow measurement of gastric residuals, and are less likely to become clogged. Moreover, since a correctly placed nasogastric tube does not worsen dysphagia, patients without high risk for aspiration may receive a limited amount of oral food, as part of early swallowing therapy, with a nasogastric tube in place [41]. However, many patients find nasogastric tubes uncomfortable and remove them leading to treatment failures and aspiration pneumonia. In these instances, use of a retention system or nasal bridle has been associated with fewer displaced tubes [8]. PEG is used in patients who remain unable to swallow after a few weeks from stroke onset. PEG is associated with fewer tube failures, is more effective than feeding via nasogastric tube in sustaining the nutritional status, and potentially reduces the risk for aspiration pneumonia [39]. However, PEG placement is an invasive procedure and can be rarely complicated by peritonitis or bowel perforation [8, 42]. In addition, the optimal timing of PEG placement is unclear. In the FOOD trial, patients with acute stroke and dysphagia who underwent PEG during the first 1-2 weeks after acute stroke had worse outcomes than patients who were fed through a nasogastric tube [43]. Enteral access is usually best guided by considering whether it will be required short term or long term. The extent and severity of the stroke as well as the need for stay in an intensive care unit play a role in the decision making. Patients with a National Institutes of Health stroke scale (NIHSS) score >16 without evidence of aspiration pneumonia or with a NIHSS score >12 and evidence of aspiration pneumonia may require early PEG placement [39]. However, neither nasogastric nor PEG tube feedings eliminate the risk of aspiration pneumonia [44].

Once a tube is placed, healthcare practitioners must make careful decisions related to monitoring the delivery and adequacy of nutritional content while monitoring for potential tube-related complications [36]. During rehabilitation, tube feeding appears to increase the risk for complications but this increase is primarily related to the severity of the underlying stroke rather than tube use per se [44]. Tube-related adverse events include aspiration, mechanical tube clogging, drug-nutrient interactions, and gastric intolerance due to formula contamination [32]. Tube feeding can also adversely affect quality of life, due to medical complications, intolerance, and the use of restraints to avoid removal of the feeding tube. The incidence and severity of tube-related complications can be reduced by selecting an appropriate enteral formula, screening clinical status and nutritional needs, and careful monitoring of the feeding process [36].

Many stroke patients require enteral feeding for several years or for their whole life. Polymeric formulas are frequently used in long-term tube-fed patients and are generally well-tolerated [20]. These formulas are comprised of intact protein, complex carbohydrates, long-chain fats, and some medium chain triglycerides (MCT). A polymeric, high-protein enteral formula that provides 1–1.5 kcal/mL is appropriate [8]. Modular products (e.g., protein powder, glucose polymers, or MCT oils) can also be added to polymeric formulas to increase either the energy density or protein content of that specific formula [36]. In addition, some formulas contain additional fiber that exerts a beneficial effect on the microbiota. When a high-fiber formula is used, adequate fluid intake is necessary to prevent constipation [36]. It should be mentioned that mixing medications with formulas should be avoided and the tube should be flushed with 30 mL of water before and after the medication is given [20, 36]. These precautions are necessary in order to prevent feeding tube obstruction and to avoid drug-nutrient interactions, which may affect drug or nutrient stability, absorption, and bioavailability leading to reduced drug efficacy, increased risk for drug toxicity, and impaired gastrointestinal tract function [32].

As swallowing difficulties improve, dysphagic patients are candidates for returning to oral feeding [28]. A large percentage of stroke patients with a feeding tube will return to oral feeding within 3 months of stroke onset [8, 45]. Therefore, feeding tube use is often temporary and reinitiating oral feeding is an important feature of clinical care of these patients [28]. Through clinical assessment of swallowing abilities and the appropriate diet modifications for dysphagia, many patients will be able to resume oral feeding [8, 28]. Frequent evaluation of tube-fed patients by a speech-language pathologist is necessary to identify subtle improvements in swallowing function that can permit the gradual transition from tube to oral feeding. At a minimum, candidates to return to oral feeding must demonstrate a safe and efficient swallow on a consistent basis and must be able to consume adequate oral food or liquid to satisfy their nutritional requirements [28]. The ability for adequate oral intake is also affected by other factors including the level of consciousness, the presence of depression and vision impairment, and arm strength [8]. Depending on patient's ability, the transition should begin with an order to stop the tube feeding for 1 hour prior to each meal. Continuous tube feedings should be gradually modified to an intermittent schedule to stimulate appetite, and the patient should be given 5 to 6 small oral feedings a day with the appropriate consistency. The choice of food to reinitiate oral feeding is complex and is based on findings from both the clinical and instrumental swallowing examinations. If a patient is able to consume orally 75% or more of the nutrition requirements consistently for 3 days then tube feeding is discontinued. During transition to oral feeding, close monitoring of the swallowing ability, hydration, electrolyte balance, body weight, and development of respiratory complications is necessary [8]. Calorie intake counting is also recommended because patients with stroke might suffer from memory deficits and might be unable to report oral intake accurately [8, 36]. The timely initiation of oral feeding after sufficient preparation reduces the incidence of chest infection, the length of hospitalization, and the mortality risk [5]. Interestingly, studies showed that undernourished patients who receive intensive oral nutritional supplementation with a high calorie, concentrated supplement during stroke rehabilitation show greater improvement in motor function and higher rates of returning home than patients on standard nutritional supplementation with a more dilute supplement of the same volume [46].

For some patients, the transition from tube to oral feeding can be challenging both physically and mentally [36]. Learning to eat again after receiving no oral intake for a long time can be tiring and distressing. Some patients may be too aggressive when returning to oral diet and experience failures which discourage them from making more attempts. Weaning requires a multidisciplinary strategy involving the physician, nurse, speech-language pathologist, and dietitian. The weaning process, goals, and plan of action should be discussed thoroughly with the patient and family members [8, 28].

## 8. Conclusions

Malnutrition is frequently observed in patients with stroke and is a risk factor for adverse outcomes. Early recognition of malnutrition is crucial but is hampered by the absence of valid markers of malnutrition. In addition, despite the considerable expenses devoted to improving nutritional status, it is unclear whether this improvement will translate into better outcomes. More studies are needed to identify the patients with stroke who will benefit more from nutritional interventions and to clarify the optimal timing and method of nutritional supplementation.

## Figures and Tables

**Table 1:** Micronutrients and mechanisms through which their deficiencies induce cerebrovascular alterations and increase the risk of stroke.

Micronutrient	Mechanism
*Folic acid *	
Cofactor in homocysteine metabolism	Hyperhomocysteinemia (potentially atherogenic)

*B vitamins *	
(i) B_6 _ and B_12_: cofactors in homocysteine metabolism	(i) Hyperhomocysteinemia (potentially atherogenic)
(ii) Potentially antioxidants	(ii) Oxidative stress

*Vitamin D*	
(i) Controls parathormone levels	(i) Secondary hyperparathyroidism:
(ii) Suppresses cholesterol uptake by the macrophages and foam cell formation	– Insulin resistance and pancreatic b-cell dysfunction→type 2 diabetes mellitus
	– Activation of the rennin-angiotensin-aldosterone system→hypertension
(iii) Increases the size of high-density lipoprotein particles	– Stimulation of systemic and vascular inflammation→atherogenesis
	(ii) Atherogenesis

*Vitamins A, C, and E *	
Antioxidants	Oxidative stress

*Zinc *	
(i) Activates brain protein synthesis	(i) Neurocognitive impairment
(ii) Controls newly formed synapses	(ii) Impaired neurotransmission
(iii) Cofactor of superoxide dismutase	(iii) Oxidative stress

**Table 2:** Indications for enteral nutrition support in stroke patients.

Indications for enteral nutrition support
Dysphagia
Inadequate nutritional intake due to
(i) Reduced consciousness level
(ii) Depression
(iii) Poor oral hygiene
(iv) Xerostomia
(v) Reduced mobility
(vi) Arm or facial weakness
(vii) Fatigue
(viii) Vision, speech, and language impairment
(xi) Cognitive deficits
Increased metabolic demands
